# Efficacy of a combined diving and mindfulness program on emotional eating in adults with obesity: Randomised controlled trial with standard care

**DOI:** 10.1371/journal.pone.0345784

**Published:** 2026-03-27

**Authors:** Karolina Griffiths, Thibaut Markarian, Vincent Meurice, Mélanie Muzellec, Fanny Lannoy, Frederic Beneton, Pierre Michelet

**Affiliations:** 1 Department of General Practice, University of Montpellier, Montpellier, France; 2 Desbrest Institute of Epidemiology and Public Health, University of Montpellier, Montpellier, France; 3 Primary Care Centre, Les Cevennes, Montpellier, France; 4 Department of Emergency Medicine, Timone University Hospital, Marseille, France; 5 UMR1263 Center of Cardiovascular and Nutrition Research (C2VN), Aix-Marseille University, INSERM, INRAE, Marseille, France; 6 BATHYSMED, Bouillante, France; CESUMAR: Centro Universitario de Maringa, BRAZIL

## Abstract

**Background:**

Emotional eating is considered as a mediator between depression and obesity. Combining mindfulness and sport activities can improve obesity-related psychological disorders but inconsistencies in effect and duration have been reported. We aimed to evaluate the effects of a combined mindfulness-diving Bathysmed® program on emotional eating scores in participants with obesity.

**Methods:**

This unblinded randomized controlled trial was performed in the community setting, Montpellier, France. Adults with a BMI > 30 kg/m2 were randomly assigned (1:1) to a Bathysmed® 2-month program (intervention group) therapeutic scuba-diving protocol with mindfulness exercises plus standard care, or stand-alone standard care including dietary and psychological support (control group). The primary outcome was the mean change in the emotional eating subscale of the *Dutch Eating Behaviour Questionnaire* (DEBQ-EE) at 2-months as intention to treat.

**Findings:**

Between July-August 2022, 63 participants were randomised, with 31 in the intervention group. One participant from each group were excluded. Fifty-five (87·3%) were female, median age 46 [35.5;54] and BMI 35.46 [33.45;39.48]. There was a statistically significant reduction of DEBQ-EE at 2 months in the intervention group (−0·82 (SD 0·81)) versus control group (−0·27 (SD 0·61) p = 0·004). This effect was maintained at 5 and 8-month follow up. There was a significant reduction in weight self-stigma, stress, and quality of life change scores in the intervention group.

**Interpretation:**

This combined diving and mindfulness program effectively reduces emotional eating, self-stigma and quality of life scores in participants with obesity, with sustained benefits. Health policies should integrate physical activity and psychological therapies for obesity management.

**Trial registration:**

Clinicaltrials NCT06882200.

## Introduction

The World Health Organisation considers obesity to be a chronic disease with multifactorial causes and one of the most common risk factors for non-communicable diseases [1]. There are an estimated 2 billion people worldwide with obesity. Alongside pharmacological and surgical options, psychological treatments are considered an essential component of obesity management. Indeed, rather than just considering obesity to be a physical and nutritional problem, it is important to consider the mental health aspects of obesity [2]. Stress and depression can negatively impact eating behaviours and weight regulation [3] and are associated with reduced quality of life [4]. Emotional eating is the disinhibition of dietary restrictions generated by stress and negative emotions [2]. Emotional eating is linked to weight fluctuations, eating disorders and is considered the mediator between depression and obesity [2].

“Weight bias” is a term to explain negative attitudes towards people who are perceived to be overweight. Prejudicial attitudes and negative stereotypes can lead to stigmatization in daily life, for example through the media, and social exclusion due to their weight. WBI is an important cause of emotional eating [5] and is part of the vicious cycle of obesity, with an increase in negative emotions in relation to their weight and reduced motivation to adopt behavioural changes [3,6] The prevalence and negative effects of weight-related stigma on health are well documented [7,8]. The internalization of this bias, or self-stigma (WBI, weight bias internalisation) is strongly associated with negative health outcomes [5].

A multidimensional approach, not solely linked to weight loss, is needed to manage patients with obesity. This should include psychological therapies combatting stress and emotional eating combined with practical techniques to increase physical activity. This will help break the vicious cycle of obesity and improve the long-term success of bariatric surgical procedures.

Meditation or mindfulness may have a positive impact on emotional eating and stress [9]. Mindfulness is commonly defined as a non-judgmental, present-moment awareness (self-regulation of attention) and acceptance of one’s ongoing experience (orientation to experience) [10]. Mindfulness-based interventions are structured, skills-based programmes designed to cultivate self-regulation by enhancing awareness of emotional and sensory cues, a mechanism considered central to modifying eating behaviours. In the field of obesity and emotional eating, a variety of mindfulness-based approaches have been used, including mindfulness-of-breath practices, mindful-eating programmes, acceptance-based therapies, and combinations with cognitive behavioural techniques [11].

Regular physical activity has indisputable benefits for physical and mental health [12–14]. Physical activity can have a positive impact on emotional eating, but the type of activity must be deemed accessible for patients with obesity. Scuba diving combines elements of physical activity and mindfulness, centering on the diver’s respiratory capabilities [15]. It has the advantage of the feeling of weightlessness underwater for patients with obesity. Previous literature has demonstrated the positive impact of diving on psychological functioning, including increased well-being in participants with physical injuries, improved parasympathetic activity and positive emotional effects [16,17]. Programs have been developed that combine diving experience and psychoeducation with mindfulness and sports mental preparation, including breathing techniques [15]. Research indicates favourable outcomes for mindfulness skills, persistent stress levels and post-traumatic stress disorder (PTSD) [13,18]. Daily diving over a week improves the level of perceived stress, mood and well-being and the effect is maintained after one month [13].

The aim of the study was to evaluate the effects of a joint mindfulness- diving program on emotional eating scores at 2 months in obese participants in comparison to the control group with standard care.

## Methods

### Study design

In this randomized controlled trial, participants with obesity were randomised to a 2-month therapeutic scuba-diving program with mindfulness exercises plus standard care, or stand-alone standard care. Participants were recruited from a primary care centre with a special interest in obesity in Montpellier, France, with a multidisciplinary team including general practitioners, psychotherapist and dietician. The overall length of the study was 10 months, July 2022- April 2023. Participants were recruited from July 01, 2022 to August 31, 2022, followed by two months of the diving program and follow-up was completed April 2023. The study was conducted in accordance with the Declaration of Helsinki (1964) and was conducted with the participants’ understanding and written consent. The study was registered and approved by the national research ethics committee in France BTY-2022-05-OBEDIVE before participation inclusion, which provided a EUDRACT number (2022-A01082-41). However, at the time of initiation, no public online registration URL was available. The study was conducted in accordance with the predefined protocol and adhered to CONSORT guidelines (Supporting Information) to ensure methodological transparency and rigor. The trial has since been retrospectively registered in Clinicaltrials.gov (Registration ID: NCT06882200). The authors confirm that all ongoing and related trials for this intervention are registered. This randomized controlled trial is accompanied by a qualitative study on the lived experience of the participants in the BathysMed group [19]. The individual in the supporting information has given written informed consent (as outlined in PLOS consent form) to publish these case details and image.

### Participants

Inclusion criteria were participants with obesity (Body Mass Index BMI > 30 kg/m2), adults over the age of 18 able to provide written consent and affiliated to public medical insurance. Non-inclusion criteria included: BMI > 45 kg/m2, older than 60 years, unable to walk, known psychiatric pathology apart from anxiety and depression, intellectual deficit, routinely practising meditation, a known contra-indication to scuba-diving or known past medical history of cardiac problems. Participant characteristics (age, sex, past medical history, medication) were self-reported or measured (BMI) at inclusion. Participants gave their written consent.

### Randomisation and masking

Participants were randomly assigned to either the intervention or control group. Two variables (sex and BMI) were known to be correlated to a higher DEBQ-EE score [20]. Dynamic adaptive stratification 1:1 randomisation was used to minimise the differences in sex and BMI between the intervention and control groups [21]. The computer-generated randomisation allocation sequence was performed using the statistical software program R and programmed by the data manager not involved with enrolment. No other staff had access to the randomisation sequence. The principal investigator and all study personnel involved in the collection and review of outcomes were blinded to group assignments.

### Procedures

#### Intervention group.

A scuba-diving training team has designed a mindfulness-associated diving program with trained instructors called the Bathysmed® program [22]. The Bathysmed program is a unique scuba diving program that combines scuba immersion with techniques from mindfulness meditation, psychology and sports mental preparation. The program included 30 different exercises over ten scuba-diving sessions with mindfulness training, twice a week for five weeks between September and October 2022. This schedule was chosen to balance psychological engagement with feasibility and recovery time for working age adults. The first two sessions in a swimming pool in Montpellier, France had a max depth of 1.5m, with the following sessions in the sea near Marseille, France at a depth of 6m, with a few deeper descents to 20m. The water temperature was between 14–20°C. On average there were 2 divers for each program instructor. Diving instructors trained in the Bathysmed® protocol guided the sessions, which were structured as follows:

- Diving theory and safety: Covering essential diving knowledge and safety measures.
- Stress and emotion theory: Discussing the understanding of stress, emotions, and reassuring behaviors, along with the underlying mechanisms of pathology to better manage it.
- Introduction to diving exercises: Addressing underwater communication challenges in advance.

Mindfulness and sophrology practice on land: Conducting exercises on land to be repeated underwater. Sophrology, developed in the 1960s by neuropsychiatrist Alfonso Caycedo, is a mind–body method combining controlled breathing, dynamic relaxation, and positive visualization to harmonize body and mind. Inspired by yoga, Zen, and phenomenology, it enhances self-awareness and stress regulation and is mainly practiced in Europe. Each session begins with mindfulness exercises designed to anchor participants in the present moment by focusing on bodily sensations and breathing rhythm. The participants first work on re-appropriating their bodily sensations, then on their mental capacities, such as concentration and attention.

Each diving session (<20 meters, 20–30 minutes)consisted of three phases:

- A static phase involving exercises to achieve a modified state of consciousness. Targeted work focusing on either the body, mind, or mindfulness levels. This is followed by dynamic exercises aimed at improving body schema representation, and by guided visualizations focusing on sensations related to hunger, satiety, digestion, impulsive eating, and craving regulation. The underwater visualizations enable participants to better perceive states of satiety and hunger, to re-register natural physiological sensations and reduce emotion-related hunger impulses. This immersive, weightless work establishes the participant’s focus on the present moment. Self-awareness, sensations and somaesthetic perceptions are heightened, and ventilatory control stimulates cardiac coherence.
- A concluding exploration phase with a gradual return to the surface.
- Debriefing of experiences: Reflecting on participants’ feelings post-dive. The program concludes with work on mindfulness and self-confidence

Reviviscence-maintenance is an automated function that keeps the breathing system active and ready during periods of non-use. In order to prolong the effect without requiring the continuation of diving sessions, reviviscence-maintenance sessions were offered from the third week with the use of a virtual reality mask and the visualization of one of the participant’s own dives (three to five different films were made), associated with a sound reproduction of the participant’s breathing which will simulate his own breathing but perceived as if he were underwater (Supporting Information). Participants were advised to use the VR masks two to three times a week for the last five weeks of the intervention program. All participants in the intervention group also received the same standard care as the control group.

#### Control group.

The control group received standard care included regular appointments and health education with their general practitioner and monthly appointments with the dietician and psychotherapist at the primary care centre in Montpellier, France throughout the study. All participants attended monthly sessions on diet, exercise and general stress management with a registered dietician. They analysed eating behaviours and identified and improved knowledge on emotional eating. Advice included recipes and how to identify their own needs and challenges. All types of “diets” were avoided. Monthly sessions with a psychotherapist were provided if indicated upon medical advice. These sessions included therapy to understand the participant’s relationship with food, particularly the mechanisms in place from childhood, to identify the moments and reasons when excessive food intake occurred, and to work on behavioural changes. Both the dietician and psychotherapist included advice on stress management including identifying external and intrinsic factors affecting them (factors leading to emotional eating) and regular discussions on how to relieve stress. No mindfulness sessions were performed.

### Primary measure

#### Dutch eating behaviour questionnaire.

The primary measure was the mean change in the emotional eating subscale of the *Dutch Eating Behaviour Questionnaire* (DEBQ-EE) at the end of the two-month protocol. This questionnaire is internationally renowned and has been translated into 15 languages, including French [23]. It has been validated with good internal validity and reliability scores by the European Federation of Psychologists Association (EFPA) [2]. The questionnaire addresses three dimensions of eating: emotional eating (DEBQ-EE), restrictive eating (DEBQ-RE) and external eating (DEBQ- ExtE), eating for or due to external factors.

#### Secondary measures.

Secondary measures were collected at baseline and at the end of the two-month protocol and at five-month and eight-month follow-up. Secondary measures included the DEBQ-RE and DEBQ-ExtE scores. Other secondary measures included weight (kg) [24], BMI (kg/m^2^) and waist circumference (cm). Reports of adverse events were collected. Further secondary measures are detailed below.

#### Weight self stigma questionnaire.

Weight bias internalisation and weight self-stigma were assessed using the Weight Self-Stigma Questionnaire (WSSQ), a validated instrument measuring self-directed stigma related to body weight [5,25,26].

#### Quality of life.

Quality of life specific to obesity was evaluated using EQVOD (*Échelle de Qualité de Vie Obésité et Diététique*), a questionnaire designed to capture the impact of obesity on daily functioning and well-being [27].

#### Perceived stress scale of cohen score.

Perceived stress was measured using the Perceived Stress Scale (PSS), which assesses the degree to which individuals appraise their life situations as stressful [24].

### Statistical analysis

The sample size calculation was based on a previous study demonstrating the benefits of mindfulness on emotional eating with the same primary outcome (DEBQ-EE) [28]. The mean change in the control group was + 0.09 (SD 0.72), compared to the experimental group, −0.44 (SD 0.70). Based on these results, we hypothesised that the Bathysmed program would reduce the mean change in DEBQ-EE scores in comparison to the control group. We estimated a sample size of 31 participants in each group, based on a power of 80% and a statistical significance level of 5%.

Descriptive statistical analysis was performed, with quantitative variables expressed as the mean and standard deviation, or median and interquartile range, and qualitative variables expressed as the number and percentage. The normality of data distribution was tested using the Shapiro-Wilk test. The intervention and control groups were compared using univariate tests (t-test, Kruskall-Wallis). Type II ANCOVA controlling for baseline scores were performed to test for differences between the two groups at each time point. T-test and ANCOVA assumptions were verified through skewness and kurtosis. ANCOVA’s assumption of homogeneity of variance (Levene’s test) and homogeneity of regression slopes were also tested. The mean changes from baseline at 2 and 5 months were compared using univariate tests (t-test or Kruskall-Wallis test). We analysed intervention effects using linear mixed models [29]. We entered group, time, group*time interaction, and the covariate age as fixed effects, and the participant identification as a random effect. Regression coefficients were used as measures for effect size. Statistical analyses were performed as intention to treat, with an additional paradigm to analyse the data using multiple imputation (MI). We imputed continuous variables using predictive mean matching, with the mice (Multiple Imputation by Chained Equations) R package [30]. The imputation model included all outcomes (baseline and follow-up data), time and control variables (age). All analyses were performed using the software program R version 4.2.2 (2022-10-31).

## Results

**Fig 1:**
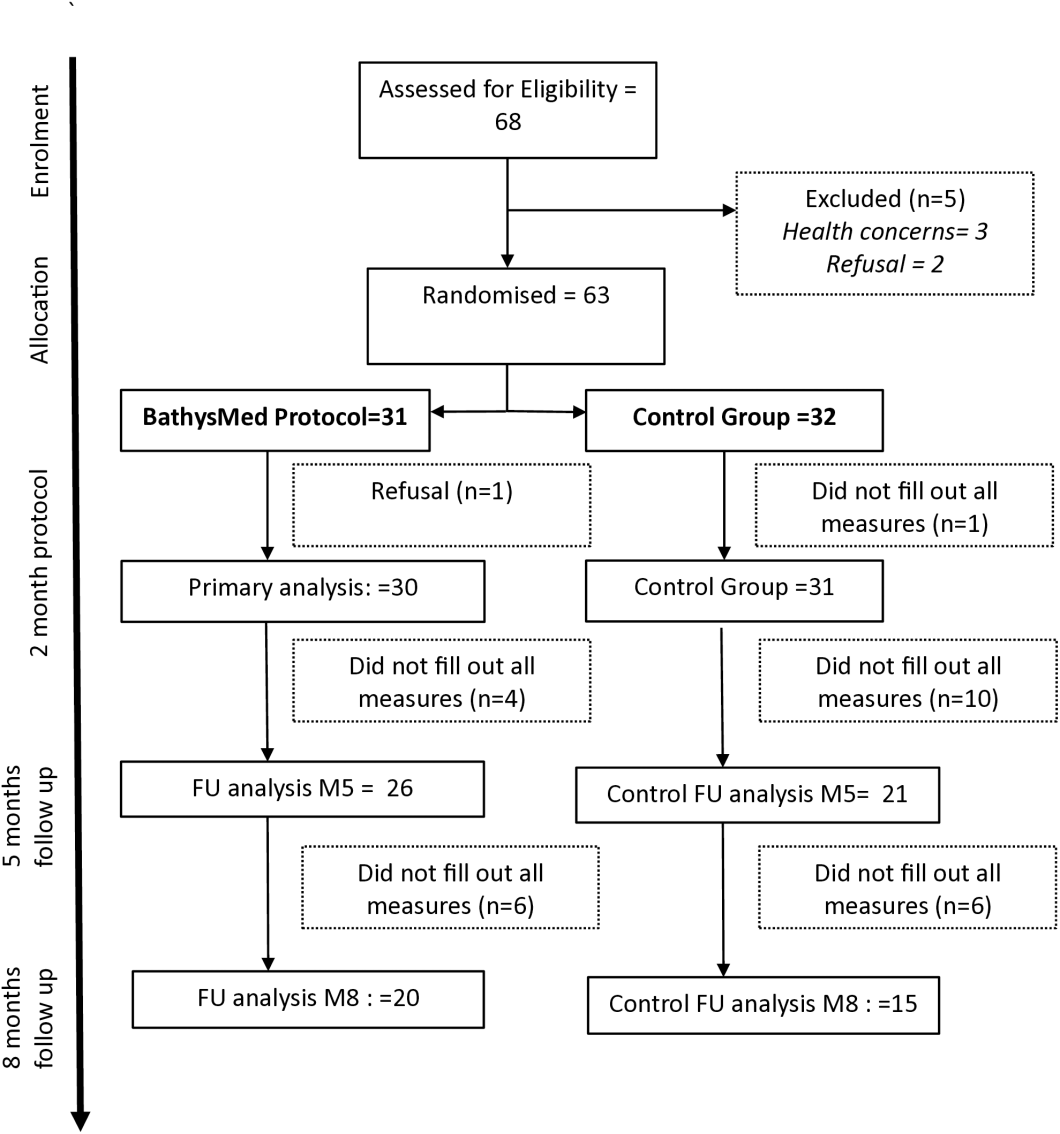
Consort Flow Diagram.

Sixty-eight participants with a BMI > 30 kg/m2 were assessed for eligibility. Three participants were excluded from the study due to health concerns (ocular tumour, uncontrolled hypertension, cardiomyopathy) and two refused to participate. Sixty-three participants were therefore randomised, with 31 (49%) in the intervention group and 32 in the control group (Fig 1). Participants in the diving group attended an average of 8·9/10 sessions. Twenty-two (71%) participants in the intervention group completed all diving sessions, accounting for 277/310 (89%) of the total required sessions in the two-month program. Four participants had diving qualifications, four had previously dived once and the other participants did not have previous diving experience. Three participants required custom-made wetsuits, the others were in standard XXXL equipment, with weights of 5–14 kg depending on size and experience. One participant did not adhere to the BathysMed program, and one participants did not complete primary outcome measures. The number of participants with missing reported primary outcome measures for the follow-up analyses was n = 5, 16·1% in the intervention group, 11 (34·4%) in the control group at 5-month follow up and 11 (35·5%) and 17 (53·1%) respectively at 8 months follow up (Fig 1).

Fifty-five (87.3%) of the participants were female, with a median age of 46 years (IQR 35.5–54). All participants had an initial medical consultation with their general practitioner, and all were seen by the dietician. 38/63 (60.3%) had concomitant sessions with the psychotherapist or psychiatrist. Baseline characteristics are shown in Table 1. Missing data rates at follow-up were not significantly different across groups (5 months x^2^(1) = 1.88, p = 0.17, 8 months x^2^(1) = 1.33, p = 0.25). No unintended side effects were reported.

**Table 1:** Baseline characteristics.

	Control Group n = 32	Intervention group n = 31	Total (n = 63)
Female (%)	28 (87.5%)	27 (87.1%)	55 (87.3)
Age, median [IQR]	48.0 [36.5;55.2]	44.0 [35.5;52.0]	46 [35.5;54]
Height, mean (SD)	1.67 (0.09)	1.67 (0.09)	1.67 (0.09)
Weight kg, mean (SD)	104 (16.8)	99.1 (14.4)	101.5 (15.7)
BMI, median [IQR]	35.9 [34.0;40.9]	34.4 [32.5;37.8]	35.46 [33.45; 39.48]
Waist circumference cm, median [IQR]	118 [111;128]	113 [106;120]	116 [108;123.5]
*Baseline scores (mean, SD)*			
DEBQ EE	3.24 (0.86)	3.61 (0.89)	3.42 (0.52)
DEBQ Ext	2.92 (0.46)	3.24 (0.54)	3.08 (0.52)
DEBQ RE	2.69 (0.72)	2.95 (0.72)	2.82 (0.72)
WSSQ	42.9 (7.91)	41.5 (6.82)	42.17 (7.37)
EQVOD	61 (13.3)	58.2 (9.48)	59.66 (11.57)
PSS	29.3 (9.91)	33 (8.31)	31.16 (9.28)

DEBQ EE: Emotional eating subscale of the Dutch Eating Behaviour Questionnaire (DEBQ), DEBQ-RE: restrictive eating subscale and DEBQ- ExtE, External eating subscale of the DEBQ, DEBQ, WSSQ: Weight Self Stigma Questionnaire, EQVOD: evaluation of quality of life (*l’échelle de Qualité de Vie Obésité et Diététique*; PSS: the Perceived Stress Scale of Cohen score.

Data are n (%), n/N (%), mean (SD); median [interquartile range].

Table 2 compares the changes from baseline for primary and secondary outcomes in each group. Overall group*time interactions, for both the ITT paradigm and for imputed data were calculated. Sensitivity analyses on absolute scores with ANOVA controlling for baseline scores are shown in Supporting Information, with similar results at 2 months.

**Table 2:** Changes from baseline in psychological, self-stigma quality of life and stress scores.

Variable	Control group, standard care			Intervention BathysMed Diving Protocol			Overall group*time effect (complete case intention to treat analysis) §	Imputed data
	2 month mean (SD) n = 31	5 month	8 month	2 month	5 month	8 month		
**DEBQ EE**	−0.27 (0.61)	−0.40 (0.80)	−0.37 (0.82)	−0.82 (0.81) **	−0.87 (0.71) *	−0.92 (0.70) *	**F = 4.84, p = 0.003**	**F = 5.00, p = 0.002**
DEBQ RE	0.00 (−0.3; 0.10)	−0.10 (0.45)	−0.19 (0.47)	−0.10 (−0.6; 0.07)	−0.18 (0.66)	−0.32 (0.61)	F = 0.39, p = 0.75	F = 1.65, p = 0.18
DEBQ ExtE	−0.10 (0.33)	−0.10 (−0.3; 0.00)	−0.18 (0.56)	−0.46 (0.40)***	−0.55 (−0.88; −0.20) ***	−0.43 (0.44)	**F = 6.14, p = < 0.001**	**F = 4.93, p = 0.002**
WSSQ Total	−3.00 (−6.00; 1.00)	−3.00 (4.90)	−1.87 (6.35)	−5.50(−8.75; −1.00) *	−7.08 (6.61) *	−6.45 (6.11) *	**F = 3.65, 0.014**	**F = 4.27, p = 0.006**
EQVOD (Total)	−0.05 (8.00)	−0.56 (−5.00; 5.00)	2.48 (10.6)	8.85 (9.16) ***	7.50 (1.53; 11.7)	7.75 (9.86)	**F = 4.91, p = 0.003**	**F = 4.70 p = 0.004**
PSS	0.00 (−4.00; 2.75)	0.19 (9.12)	−2.67 (5.73)	−4.50 (−15.00; 2.00) **	−6.54 (10.8) *	−8.65 (10.8) *	**F = 5.03, p= < 0.001**	**F = 3.91, p = 0.01**
Weight	−0.20 (−0.85, 0.95)	−0.02 (2.30)	0.00 (−1.20; 2.10)	−0.40 (−2.70; 0.00)	−1.27 (3.89)	0.00 (−4.40; 3.40)	F = 1.93, p = 0.13	**F = 2.70, p = 0.0473**

* p < 0.05.

** p < 0.01.

***p < 0.001.

DEBQ EE: Emotional eating subscale of the Dutch Eating Behaviour Questionnaire (DEBQ), DEBQ-RE: restrictive eating subscale and DEBQ- ExtE, External eating subscale of the DEBQ, DEBQ, WSSQ: Weight Self Stigma Questionnaire, EQVOD: evaluation of quality of life (*l’échelle de Qualité de Vie Obésité et Diététique*; PSS: the Perceived Stress Scale of Cohen score.

§ Extracted from Type III ANOVA tables of the regression models assessing intervention effects, under complete cases with intention to treat analysis. The F values correspond to the treatment * time interaction.

Bold represents statistically significant results considering p < 0.05.

**Fig 2:**
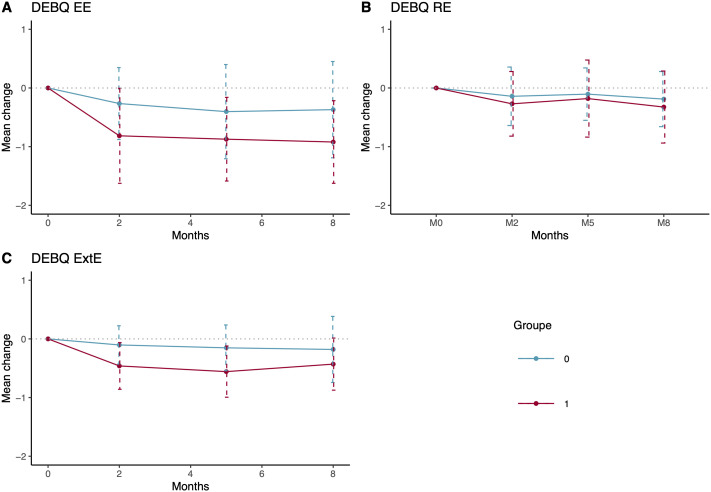
Mean change in DEBQ subscale scores from baseline, at 2, 5 and 8 months. DEBQ EE Emotional eating subscale (Primary Outcome) **(A)**, DEBQ Restrictive eating **(B)**, DEBQ External eating **(C)**.

Fig 2(A) shows the mean change in the DEBQ-EE score (the primary outcome) between baseline and 2-months. In univariate analysis, there was a statistically significant reduction in the intervention group −0.82 (SD = 0.81) in comparison to the control group −0.27 (SD = 0.61), p = 0.004. This effect was maintained at the 5 and 8-month follow-up. The Group*time effects in both analysis paradigms supported these findings of significant differences between groups over time.

There was a greater reduction in the external eating scores at 2 months in the intervention group −0.46 (SD = 0.4) than in the control group (M = −0.10, SD = 0.33, p < 0.001) (Fig 2 (B)). This effect was maintained at 5 months (p < 0.001) but not at 8 months (p = 0.168). There was no significant difference in the change in restrictive eating subscale of the DEBQ (Fig 2C).

**Fig 3:**
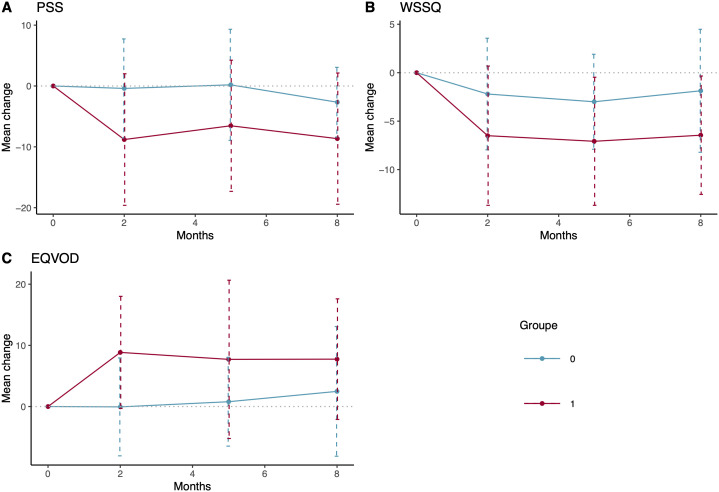
Mean change in secondary outcomes from baseline at 2, 5 and 8 months. PSS Perceived stress scale **(A)**, WSSQ, Weight self-stigma questionnaire (B) and EQVOD, Obesity and Diet Quality of life scale **(C)**.

Weight self-stigma scores decreased at 2 months, with a significantly lower change in the intervention group (M = −5.50, [IQR −8.75; −1.00]) in comparison to the control group (M = −3.00, [IQR −6.00;1.00], p < 0·001), maintained at both 5 months (p = 0·002) and 8 months (p = 0.040) follow up. (Fig 3).

Quality of life scores on the EQVOD demonstrated a significant improvement from baseline to after the 2-month program in the intervention group (M = 8.90, SD = 9.19) in comparison to the control group (M = −0.13, SD = 8.10, p < 0·001), but not at follow up (Fig 3).

The perceived stress score was statistically significantly different at all time points during the study.

Average weight remained stable between groups from baseline to post-2-month protocol (Table 2). Measures were also stable during the same time period for BMI (mean change for intervention group −0.10, [IQR −0.42; 0.31] and control group −0.16 [IQR −1.22; 0.00] respectively, p = 0.19) and waist circumference (mean change intervention group −0.00 [IQR −1.00; 0.00] and control group −0.00 [IQR −2.75; 0.00] respectively, p = 0.27. Measurements at the 5- and 8-month follow-up were also stable for weight, BMI and waist circumference. Overall group*time effects of the linear mixed model for weight changes did not suggest any differences between groups for intention to treat analyses but suggested a difference between groups on imputed data.

Post-hoc exploratory analyses were conducted to explore the benefits of the virtual reality (VR) masks on DEBQ-EE. Sixteen participants in the intervention group participated in the VR sessions and were compared to those who did not participate. Baseline scores were the start of the VR sessions at one month into the program. No significant difference was noted immediately post-program (M = −0.08, [IQR −0.31;0.46] versus M = −0.28, [IQR −0.48; −0.13], p = 0.072). However, the mean DEBQ-EE change from baseline was significantly different between those using masks at 5 months (M = 0.41, SD = 0.82) versus those not using VR masks (M = −0.47, SD = 0.58, p = 0.010). This effect was maintained at 8-month follow-up (M = 0.15, SD = 0.66) versus −0.51 (SD = 0.45, p = 0.03).

## Discussion

We reported the benefits of a combined diving-mindfulness program for the first time in participants with obesity. We demonstrated a significant reduction in emotional eating scores at the end of the program and maintained up to 8 months in the diving group, which is inconsistently found in the literature [28,31]. Such long-lasting effects were not demonstrated in previous studies, such as an 8-week mindfulness program [28] and a 4-month mindfulness training alongside diet and exercise guidelines [31].

Mindfulness techniques do not aim to achieve rapid weight loss, but to promote lasting changes in eating behaviour. The absence of significant change in weight, BMI and waist circumference likely reflects the short follow-up period and the fact that these were secondary outcomes, whereas emotional eating and weight stigma represent earlier targets in the behavioural change process. A meta-analysis of 11 randomized controlled trials indicated a significant reduction in the severity of binge eating through mindfulness meditation. However, the positive effect was not long-term, as it waned during follow-ups between 3 and 6 months [32]. In comparison, we demonstrated a longer positive effect from scuba diving sessions and mindfulness meditation exercises, probably due to virtual reality (VR) mask sessions. Our results suggest those using the VR mask maintained a reduction in emotional eating scores up to the 8^th^ month mark in comparison to those who did not participate in the VR maintenance sessions.

Our study significantly reduced participants self-stigma or weight bias internalisation (WBI). In the current trial, we reported an unprecedented reduction in the self-stigma score (WSSQ) at the end of the 2-month program, with the effect maintained at 5 and 8 months. Pearl et al. (2020) evaluated the effect of a 26-week program based on a cognitive-behavioural approach and showed a significant reduction in the WSSQ total score at 12 weeks, but the effect was not maintained at 26 weeks [33]. In another randomized controlled trial, the WSSQ total score was significantly reduced after evaluating a 3.5-month program based on acceptance and mindfulness in overweight or obese participants, but did not report follow-up measurements [34].

We demonstrated the benefits of health programs combining a specific sports activity and psychological health [35]. Our results seem to indicate that an original program combining dedicated diving activity and mindfulness meditation may improve stress pathologies linked to obesity more effectively than mindfulness meditation alone. We demonstrated a significant reduction in perceived stress scores at 2- and 5-months follow-up. In comparison, a randomized controlled trial showed a non-significant reduction in PSS in the intervention group of a 4-month intervention based on mindfulness meditation in overweight or obese participants [31]. Our results may be due to unique aspects of diving: better emotional regulation through the use of ample and regular breathing (cardiac coherence), strengthening of bodily anchoring and constant attention (meditation- mindfulness) [15]. These aspects illustrated how diving can be considered similar to techniques used in mindfulness meditation and were classically associated with better regulation of emotion pathways and stress, implying an improvement in regulation of the autonomic nervous system (ANS) via strengthening of the parasympathetic (vagal) system.

Our study demonstrated the synergy of mindfulness and diving. The protocol was designed in a way to increase adherence (only once a week, on a weekend), and our adherence rates to the diving program were high (89% of sessions), especially in comparison to other mindfulness programs without an integrated sports components with low adherence reported (55.8–63.4%) [36]. Our study was safe: we did not report any significant side effects from the intervention. The global effects of diving on quality of life were reported, including improved confidence and reduced stigma. The participants reported being at ease in the water, less pressure on joints, not feeling their weight under water. The lived experience of participants in the intervention group is being studied in an ongoing qualitative study. We deemed our approach to be successful in creating a lasting dynamic that reduced the sedentary behaviour of obese participants.

There were several limitations to our study. The health centre had a special interest in obesity, which may lead to participant selection bias. However, less so than secondary care-based recruitment as the centre was also a standard primary care centre with general practitioners. Participants were invited when visiting the centre and volunteered to participate in the study. Randomisation was carried out to minimise bias. The sample size was small but based on previous studies [28]. There were several exclusions and losses to follow-up at the start of the protocol, but this attrition bias had been anticipated by assessing more participants for eligibility than the estimated sample size. The main analysis was performed on an intention-to-treat basis. Loss to follow-up was high at 5 months and 8 months, consistent with similar studies on participants with obesity [36], and statistical analyses took this into account. The absence of blinding and the substantially greater contact time in the intervention arm mean that non-specific factors (e.g., attention and expectancy effects) may partly account for the observed between-group differences.

The technical conditions specific to this study may limit its generalization and reproducibility, however, the study demonstrated that new participation in diving is possible for obese participants. For example, we expected to have more technical constraints regarding adapted wetsuits, but only three were needed. No adverse outcomes were reported from diving. The ongoing qualitative study will help understand the acceptability of diving protocols.

Participants with a BMI greater than 45 kg/m^2^ or with medical contraindications to scuba diving were not able to participate in our study. Diving sessions using virtual reality masks, like those carried out in the second part of the protocol, could be offered to these participants. This could be the subject of a future study to evaluate the effect of meditative diving in virtual reality on the emotional eating of obese participants.

## Conclusion

In summary, our combined diving and mindfulness program reduced emotional eating and self-stigma, while improving quality of life scores in participants with obesity. The innovative Bathysmed® program combining sport and mindfulness promoted physical activity through scuba diving and encouraged changes in eating behaviour through mindfulness meditation.

This holistic approach went beyond weight loss goals and aimed to improve overall quality of life. This type of synergistic program confirmed the interest in developing strategies combining sports and health in primary care to combat stress and psychological health and self-stigma for participants with obesity. Future studies could analyse the application of a combined program to other chronic conditions.

## Supporting information

S1 File S1 Table CONSORT 2010 checklist of information to include when reporting a randomised trial. S2 Table: Primary and secondary outcomes at baseline, after the protocol (2 months), and follow up at 5 and 8 months. Data is presented as mean (SD standard deviation) unless otherwise specified. Type II ANCOVA adjusted for baseline scores. Statistically significant results =<0.05 are in bold. NA = not applicable. S1 Fig: Virtual reality headset BathysMed.
(DOCX)
